# Editorial: Eating Behavior Research in Children's and Adolescent's Naturalistic Environment

**DOI:** 10.3389/fpsyg.2019.02139

**Published:** 2019-09-19

**Authors:** Sandra Verbeken, Andrea Beth Goldschmidt, Catharine Evers, Caroline Braet, Lien Goossens

**Affiliations:** ^1^Department of Developmental, Personality, and Social Psychology, Ghent University, Ghent, Belgium; ^2^Department of Psychiatry and Human Behavior, Warren Alpert Medical School of Brown University, The Miriam Hospital, Providence, RI, United States; ^3^Psychology Department, Utrecht University, Utrecht, Netherlands

**Keywords:** eating behavior, children, adolescents, EMA, overeating, foodrefusal

## Purpose of the Research Topic

Children and adolescents' eating behavior is determined by many intra and extra personal factors, including individual differences in feelings of hunger and satiety, responsiveness to food, food choices and preferences, mood state, and environment/context. Advances in technology and innovations in research methodology have facilitated our understanding of how, why, and under what conditions such factors influence eating behavior in youth. The aim of this Research Topic was to compile research reports executed by experts in eating behaviors that employed naturalistic approaches. More specifically, the objective was to present results of high quality studies focusing on critical research questions, using complicated designs, and describing impactful findings that may have substantial implications for future research. This collection of papers allows reflection on important challenges researchers face with when studying eating behavior in children.

After reading this Research Topic, we hope readers will have a deeper understanding of and appreciation for how contemporary researchers use daily assessment methodologies for studying eating- and weight-related constructs in both children and adults, how modern technology can be exploited for this purpose, and the importance of studying these topics in naturalistic contexts. The compiled articles also highlight opportunities to assess antecedents as well as consequences of eating- and weight-related constructs at multiple time points, and to study community-based samples of youth, as well as clinical samples characterized by overeating or food refusal.

## Overview of Contributions

Three contributions of this topic focus on original research in community-based samples. The paper by Verschueren et al. examined the temporal sequence of eating disorder symptomatology and identity formation in a sample of high school students. In this paper, the naturalistic approach is reflected in the longitudinal design comprising three annual measurement waves, which allowed demonstration of the developmental interplay between adolescents' identity formation and the development of eating disorder symptoms. Also prospective in nature, the paper by Debeuf et al. used a daily diary design in adolescents to investigate the relationship between stress and eating behavior and the moderating roles of emotion regulation and emotional eating. Findings confirm the association between daily stress and youngster's eating behavior. The results regarding the moderating role of maladaptive emotion regulation and emotional eating did not reach significance. A third study including a community-based sample, by Ha et al., presents results from a pilot study exploring the influence of a food advertising literacy training on food choices in 8 to 13-year-old children. The training comprised four sessions of food advertising literacy training in 1 week, two of them held in the lab and two of them held at home reflecting the naturalistic element of this design. Interestingly, findings showed a reduction in the influence of taste attributes in the food decision making process of children after training. Taken together, these contributions used naturalistic designs to demonstrate that intra-personal factors such as identity formation and stress may explain youngsters' (pathological) eating behavior and that training children to cope with extra-personal factors such as food advertising may impact their food choices.

Other contributions of this topic focus on research in clinical samples. The paper by Lucarelli et al. describes the developmental course of infantile anorexia, a disorder that is recently defined as one of the subtypes of Avoidant-Restrictive Food Intake Disorder. The naturalistic approach of this paper is reflected by the four measurement points at which children and their mothers were assessed over time, more specifically, when children were ages 2, 5, 7, and 11 years. Although children's malnutrition somewhat improved over time, results demonstrated that a substantial proportion of the children (as well as their mothers) were still at a high risk of malnutrition and elevated psychopathological symptoms. Moreover, maternal factors appeared to be the best predictor of emotional/behavioral problems in the group of children who suffered from persistent malnutrition. The importance of considering a transactional view (i.e., including parental factors) when studying eating- and weight-related behavior in children is also an important theme in the commentary paper by Larsen et al. These authors elaborate on how general parenting styles may moderate the relation between weight-related parenting practices, and behaviors and weight outcomes among children. This commentary underscores the need for more research in the eating disorders and obesity fields that uses ecological momentary assessment. Finally, in the paper by Legenbauer et al., a clinical sample of females with bulimia nervosa, binge eating disorder, and controls without eating disorders were recruited. Using an ecological momentary assessment design, evidence was found for a relationship between dysfunctional eating related cognitions and binge eating disorder and bulimia nervosa thereby providing support for cognitive models on eating disorders. This study's use of innovative technology may guide similar research in youngsters. In sum, using naturalistic designs, these papers cumulatively demonstrate how intra-personal factors such as dysfunctional eating related cognitions and extra-personal factors like parenting styles, practices, and parental psychopathology may be implicated in clinical feeding, eating and weight pathology.

## Recommendations for Future Research

### The Importance of Studying Eating Behavior From a Transactional Perspective

The results of the included studies demonstrate that it is important to incorporate a transactional viewpoint when studying child and adolescent eating behavior. Although previous research in this area has already focused on examining the role of biological, psychological, and sociocultural factors in improving our understanding of eating behavior, most studies focus on one only of these levels in isolation from the others. However, Lucarelli et al. stress the importance of evaluating maternal psychopathogological symptoms which may contribute to the onset, severity, and persistence of children's disordered eating over time. Moreover, as Larsen et al. argue, more general parenting practices should be taken into account as an important moderator in the link between feeding practices and eating and weight related outcomes in children. Thus, future research may include a comprehensive approach in which factors that can be situated on different levels are examined together.

### The Importance of Taking Into Account Developmental Tasks

Next, when studying eating behavior in children and adolescents, it seems crucial to acknowledge the developmental tasks youth are facing, as well as the complex dynamic relation between development and eating problems. For example, as demonstrated by Lucarelli et al., problematic eating behavior that occurs at very young ages influences many aspects of children's development, such as emotional and behavioral functioning. These authors suggest that it is important in future research to measure the effects of malnutrition on the onset of puberty. Thus, it will be important to investigate not only how stress related to developmental tasks may influence eating behavior, but also the inverse of how eating behavior may influence development. This point is also underscored by Verschueren et al., who reported a bidirectional relation between problems with identity formation, an important developmental task during adolescence, and pathological eating behavior in adolescents.

### Additional Insights From Real Time Approaches

Although from a developmental psychopathology perspective, longitudinal studies opportunities to tease apart timing and temporal sequencing of risk factors for (maladaptive) eating behaviors, the use of real-time approaches may offer additional insights into momentary predictors or correlates of eating behavior in children and adolescents. In the study of Debeuf et al., a daily diary design showed that daily stress is associated with trajectories of eating motives over 7 consecutive days of assessment. This adds to our understanding of momentary associations between stress and eating behavior as fine-grained analysis are often lacking so far. In the study by Legenbauer et al., an EMA-approach with a signal-sampling and event-sampling design over a period of 48 h was able to demonstrate how cognitive content exerts an impact on eating behavior in daily life. Although future research should examine whether the findings in adults can be generalized to younger age groups, this study was the first to show the impact of specific patterns of dysfunctional eating-related cognitions for different ED diagnostic categories using an EMA design. Moreover, as evidence from longitudinal studies and ecological momentary assessment designs increase our understanding about how affect, cognition, and behavior interact and unfold over time, new and emerging theoretical models can be developed to inform interventions, including those delivered in real time. For example, Ha et al. targeted a sociocultural variable (food advertising) and delivered training in both a controlled laboratory environment and in the children's naturalistic environment. With their training, they were able to influence food choice behavior in children between 9 and 13 years old.

## Conclusion

We conclude that the papers of this Research Topic focus on research questions that advance the field and guide future research on which (combination of) factors may be studied to enhance insight in children and adolescent's normal and pathological eating behavior. Furthermore, the challenging research designs that are used throughout the different papers offer insight on how innovative research questions may be studied in children's and adolescents' everyday environment.

## Author Contributions

SV and LG: contribution to manuscript review and responsible for writing the first draft of the current editorial. AG, CE, and CB: manuscript review.

### Conflict of Interest Statement

The authors declare that the research was conducted in the absence of any commercial or financial relationships that could be construed as a potential conflict of interest.

